# Association Between Hemoglobin Glycation Index and Prevalence of Cardiovascular Disease in Middle‐Aged and Older Adults With Hip Fracture: Findings From CHARLS and NHANES

**DOI:** 10.1002/clc.70481

**Published:** 2026-10-07

**Authors:** Xinyu Ma, Bo Yuan

**Affiliations:** ^1^ Department of Orthopedics Civil Aviation General Hospital Beijing China

**Keywords:** cardiovascular disease, CHARLS, hemoglobin glycation index, hip fracture, NHANES

## Abstract

**Background:**

Cardiovascular disease (CVD) is a major cause of morbidity in middle‐aged and older adults with hip fracture. This study examined the association between the hemoglobin glycation index (HGI) and CVD prevalence in this population.

**Methods:**

Data were obtained from the China Health and Retirement Longitudinal Study (CHARLS) and the National Health and Nutrition Examination Survey (NHANES). Multivariable logistic regression models were used to examine the association between HGI and prevalent CVD among participants with a history of hip fracture. Sensitivity analyzes and subgroup analyzes were performed to evaluate result robustness.

**Results:**

The final analysis included 264 participants from CHARLS and 194 from NHANES. In CHARLS, the highest HGI tertile was significantly associated with higher CVD prevalence after full multivariable adjustment; a positive association was also observed when HGI was modeled continuously. In NHANES, after accounting for the complex survey design, the associations for HGI tertiles were no longer statistically significant, although a significant positive association persisted for continuous HGI. Sensitivity analyzes yielded broadly consistent patterns within each cohort.

**Conclusion:**

Elevated HGI was significantly associated with higher CVD prevalence among middle‐aged and older adults with hip fracture in the CHARLS cohort, independent of diabetes status. In the NHANES cohort, the association was attenuated and largely non‐significant after full adjustment. These heterogeneous findings suggested that HGI may serve as a complementary marker of inter‐individual glycation variation in selected populations. Given the cross‐sectional design, the results should be regarded as hypothesis‐generating; prospective studies are needed to evaluate any potential clinical utility.

**Clinical Trial Number:**

Not applicable.

## Graphical abstract

Elevated hemoglobin glycation index is associated with higher cardiovascular disease prevalence in middle‐aged and older adults with hip fracture in CHARLS, independent of diabetes. Findings were attenuated in survey‐weighted NHANES. HGI may serve as a complementary marker of inter‐individual glycation variation.

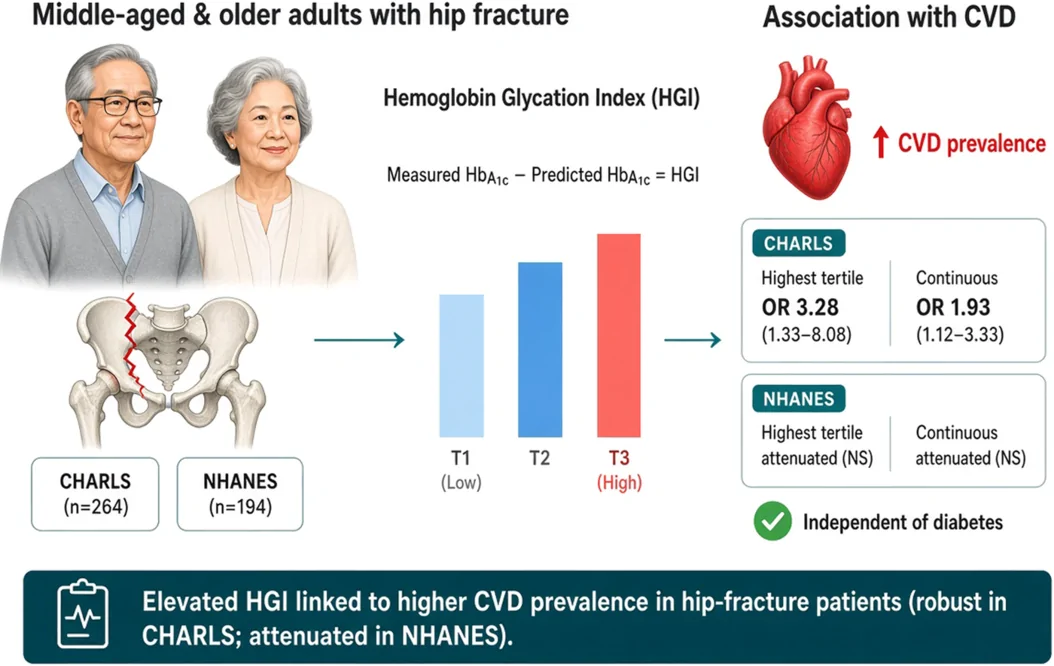

**Abbreviations**
BMI: body mass index
CHARLS: China Health and Retirement Longitudinal Study
CI: confidence interval
CVD: cardiovascular disease
DBP: diastolic blood pressure
eGFR: estimated glomerular filtration rate
FBG: fasting blood glucose
HbA1c: glycosylated hemoglobin
HDL‐C: high‐density lipoprotein cholesterol
HGI: hemoglobin glycation index
LDL‐C: low‐density lipoprotein cholesterol
NHANES: National Health and Nutrition Examination Survey
OR: odds ratio
PP: pulse pressure
SBP: systolic blood pressure
TC: total cholesterol
TG: triglyceride
VIF: variance inflation factor
WBC: white blood cell

## 1 Introduction

Hip fracture represents a major global public health challenge, particularly among middle‐aged and older adults [1]. Shen et. al reported that the fracture‐related hospitalization rate among Chinese adults was 4.39 per 1000 person‐years, with a marked increase observed with advancing age [2]. Similarity, hip fractures are highly prevalent in the United States and are associated with substantial declines in quality of life and increased mortality [3]. Therefore, comprehensive health management of aging individuals who experience hip fractures warrants particular attention.

Accumulating evidence has demonstrated that bone mineral density (BMD) was inversely linked to the prevalence of cardiovascular disease (CVD) among individuals with diabetes [4], chronic kidney disease [5] and in older populations [6], highlighting the necessity of vigilant cardiovascular risk assessment in patients with osteoporosis or fracture. Hip fracture has often been referred to as the “last bone fracture of life,” and patients sustaining a hip fracture are at a higher risk of developing CVD [3]. Moreover, the coexistence of CVD and hip fractures significantly increased mortality risk and worsened clinical prognosis [7]. Identifying independent risk factors for CVD in patients with hip fracture is therefore crucial for recognizing high‐risk individuals and optimizing personalized prevention and management strategies.

A potential interaction between glucose metabolism and osteoporosis or fracture risk has been increasing recognized [8]. The accumulation of advanced glycation end products adversely affected bone mineral density, skeletal muscle mass and bone turnover [9]. The hemoglobin glycation index (HGI), calculated as the difference between measured and predicted glycosylated hemoglobin (HbA1c) values, has been proposed as a metric to reflect individual variability in glucose metabolism [10]. Previous research have demonstrated that elevated HGI levels were significantly associated with increased CVD risk in the general population [11], in patients with diabetes or prediabetes [12] and in those with cardiovascular‐kidney‐metabolic syndrome patients [13]. Notably, HGI has been suggested to more accurately capture long‐term glycemic exposure and the tissue glycation burden than HbA1c alone, thereby complementing conventional glycemic assessment in clinical practice [14]. However, the relationship between HGI and CVD risk in middle‐aged and older patients with hip fracture remains unknown and warrants further investigation.

Given the substantial clinical and societal burden of hip fracture–related cardiovascular complications in aging populations, elucidating modifiable metabolic risk factors is of considerable importance. Therefore, this study aimed to investigate the relationship between HGI and CVD in middle‐aged and older patients with hip fractures.

## 2 Materials and Methods

### 2.1 Study Design and Populations

The China Health and Retirement Longitudinal Study (CHARLS) is a nationally representative longitudinal survey conducted by Peking University, designed to collect comprehensive data on sociodemographic characteristics, physical and mental health status, and lifestyle factors among Chinese adults aged 45 years and older through standardized questionnaires and examinations [15]. In the present study, participants with a history of hip fracture were identified from the CHARLS 2015 (http://charls.pku.edu.cn/). All participants provided written informed consent, and the study protocol was approved by Peking University institutional review board (IRB00001052‐11015). This study was conducted in accordance with the Strengthening the Reporting of Observational Studies in Epidemiology (STROBE) reporting guidelines.

The National Health and Nutrition Examination Survey (NHANES) is a nationally representative, cross‐sectional survey designed to assess the health, nutritional status and sociodemographic characteristics of the civilian, non‐institutionalized U.S. population through structured interviews, physical examinations, and laboratory assessments. In this study, participants with a history of hip fracture were extracted from seven NHANES survey cycles conducted between 2001 and 2020 (https://www.cdc.gov/nchs/nhanes/index.htm). All NHANES protocols were approved by the National Center for Health Statistics (NCHS) Ethics Review Committee, and written informed consent was obtained from all participants. This study also adhered to the STROBE reporting guidelines.

Participants from both cohorts were excluded if they met any of the following criteria: (1) age younger than 45 years; (2) missing data on fasting blood glucose (FBG) or HbA1c; or (3) incomplete questionnaires data required for the evaluation of CVD. After applying the exclusion criteria, a total of 264 patients from the CHARLS cohort and 194 patients from the NHANES cohort were included in the final analyzes. As this study involved secondary analyzes of publicly available, de‐identified data, additional ethical approval from our institutional review board was not required. The selection process for study participants is illustrated in Figure 1.

**Figure 1:**
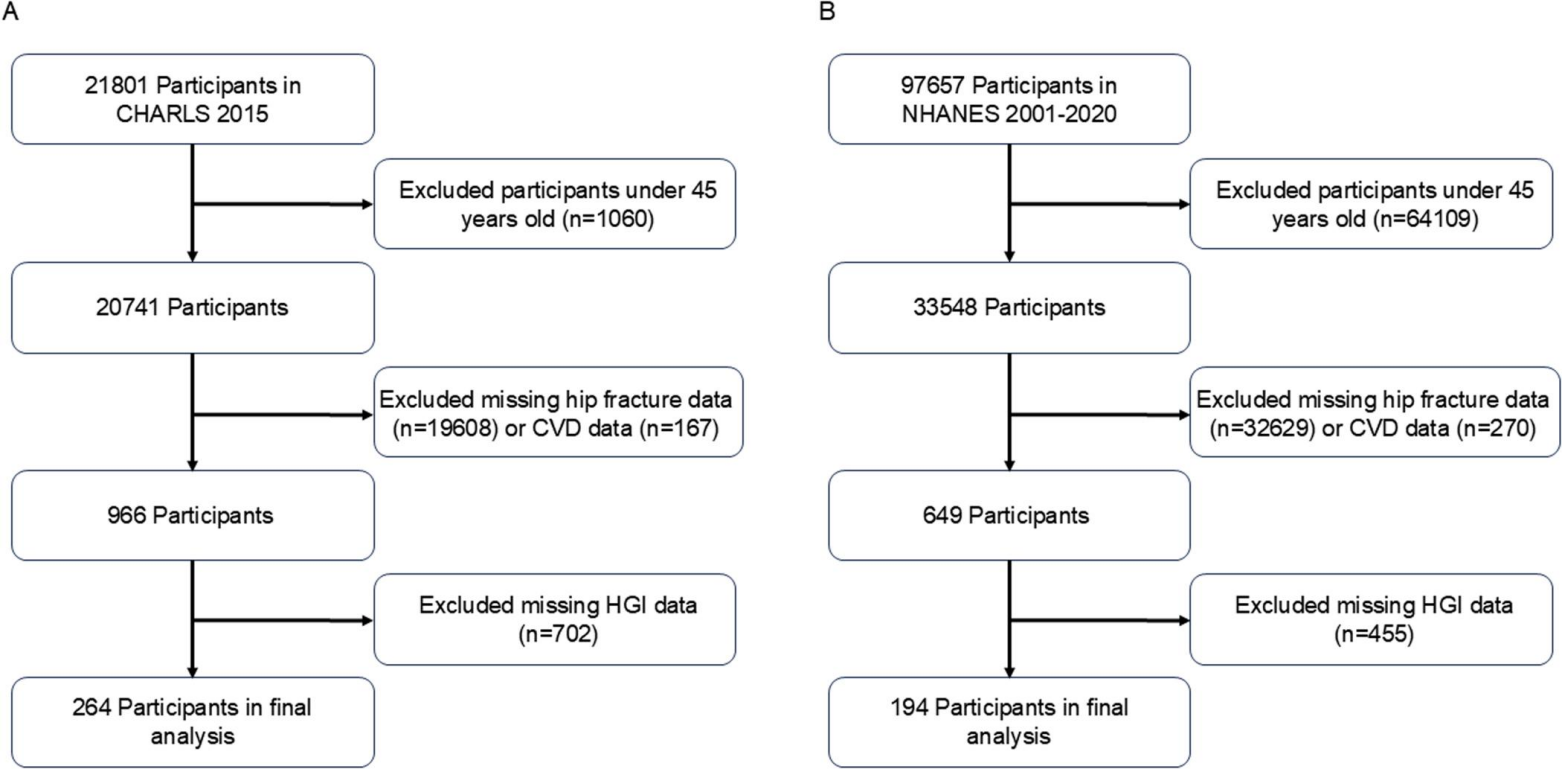
Flowchart of participant enrollment from (A) CHARLS and (B) NHANES.

### 2.2 Definition of Hip Fracture

In both the CHARLS and NHANES datasets, hip fractures status was determined based on self‐reported responses to the standardized question: “Have you ever experienced a hip fracture?” Participants who answered “yes” were classified as having a history of hip fracture.

### 2.3 Definition of CVD

In CHARLS and NHANES, the CVD was defined using a standardized self‐reported question: “Have you been diagnosed with a heart attack, angina, coronary heart disease, heart failure, or other heart problem or stroke?” for CHARLS and “Have you been diagnosed with a congestive heart failure, coronary artery disease, angina/angina pectoris, heart attack, and stroke?” for NHANES [16, 17]. Participants who responded affirmatively to any of these conditions were considered to have prevalent CVD.

### 2.4 Calculation of HGI

In this study, HbA1c and FBG levels were used to establish cohort‐specific linear regression models to estimate predicted HbA1c values within the final analytic samples of participants. The resulting equations were: predicted HbA1c = 0.327FPG + 4.137 for the CHARLS cohort; and predicted HbA1c = 0.343FPG + 3.724 for the NHANES cohort. The HGI was calculated as the difference between the measured HbA1c and the predicted HbA1c value, as previously described [13]. These cohort‐specific equations were derived from the included study populations (rather than the entire CHARLS or NHANES cohorts) to better reflect the relationship between FPG and HbA1c in middle‐aged and older adults with hip fracture. The coefficients of determination (*R* ^2^) of the linear regressions were 0.415 and 0.349 in the CHARLS and NHANES analytic samples, respectively.

### 2.5 Covariates

Baseline demographic and clinical characteristics were extracted from both databases, including age, sex, education attainment, smoking status, systolic blood pressure (SBP), diastolic blood pressure (DBP), pulse pressure (PP) and body mass index (BMI). Information on comorbid chronic conditions, including hypertension, diabetes, hyperlipidemia, cancer, chronic pulmonary disease and arthritis, was also collected. Laboratory parameters included white blood cell (WBC), hemoglobin, platelet, creatinine, total cholesterol (TC), low‐density lipoprotein cholesterol (LDL‐C), high‐density lipoprotein cholesterol (HDL‐C), triglyceride (TG), FBG, HbA1c and uric acid.

### 2.6 Statistical Analysis

All statistical analyzes were conducted using SPSS software version 25.0 (IBM Corp., Armonk, NY, USA). Continuous variables with a normal distribution are presented as mean ± standard deviation (SD), whereas non‐normally distributed variables are expressed as medians with interquartile ranges. Categorical variables are presented as frequencies and percentages. Differences in baseline characteristics between groups were assessed using the independent‐samples *t* test or the Mann–Whitney *U* test for continuous variables, and the chi‐square test for categorical variables.

In the CHARLS cohort, multivariable logistic regression models were used to examine the association between HGI (both as tertiles and as a continuous variable) and prevalent CVD. Covariates were selected a priori based on clinical relevance and evidence from previous literature regarding established risk factors for CVD in older adults. Three sequential models were constructed: Model 1 was unadjusted; Model 2 was adjusted for age, sex, and education; and Model 3 was further adjusted for smoking status, hypertension, diabetes, hyperlipidemia, and estimated glomerular filtration rate (eGFR). Multicollinearity was assessed using the variance inflation factor, with all values < 5 in the fully adjusted model. For the NHANES cohort, survey‐weighted multivariable logistic regression was performed to account for the complex multistage survey design with the same covariate adjustment strategy as in CHARLS. When combining multiple NHANES cycles (2001–2020), the original 2‐year mobile examination center weights were adjusted by dividing by the number of included cycles to construct the final analytic weights. In addition to categorical analyzes by HGI tertiles, HGI was also modeled as a continuous variable.

Sensitivity analyzes were conducted by restricting the analyzes to participants without diabetes. To address potential overfitting given the limited number of events, we performed additional sensitivity analyzes using a more parsimonious model that retained only the most essential covariates (age, sex, education, hypertension, and diabetes). Subgroup analyzes stratified by age (≤ 65 vs > 65 years) were performed, and the interaction between HGI and age was tested. A two‐tailed *p* value < 0.05 was considered statistically significant.

## 3 Results

### 3.1 Characteristics of the Study Population

In the CHARLS cohort, 264 participants with a history of hip fracture were included (mean age 64.40 ± 10.17 years; 46.59% male). As shown in Table 1, patients with CVD were significantly older and had higher SBP, lower eGFR and higher prevalences of hypertension, hyperlipidemia, and arthritis than those without CVD (*p* < 0.05). In the NHANES cohort, patients with CVD were likewise older and exhibited higher creatinine, lower DBP, platelet and eGFR, as well as higher prevalences of smoking, hypertension, and hyperlipidemia (*p* < 0.05).

**Table 1:** Baseline demographic and clinical characteristics in the CHARLS and NHANES cohort.

	CHARLS			NHANES		
	Non‐CVD (*n* = 197)	CVD (*n* = 67)	*p*	Non‐CVD (*n* = 150)	CVD (*n* = 44)	*p*
Age (years)	63.46 ± 10.30	67.15 ± 9.28	0.010	67.09 ± 12.14	71.86 ± 10.32	0.011
Male sex (%)	92 (46.7)	31 (46.3)	0.951	67 (44.7)	22 (50.0)	0.532
Education (%)			0.286			0.588
Below high school	160 (81.2)	52 (77.6)		59 (39.3)	13 (29.6)	
High school and above	37 (18.8)	15 (22.4)		91 (60.7)	31 (70.5)	
Smoke (%)	83 (42.1)	31 (46.3)	0.555	82 (54.7)	34 (77.3)	0.007
SBP (mmHg)	124.82 ± 19.9	131.66 ± 21.41	0.021	129.45 ± 19.85	126.95 ± 24.37	0.502
DBP (mmHg)	74.42 ± 12.03	76.27 ± 13.55	0.306	67.93 ± 14.79	61.00 ± 19.94	0.016
PP (mmHg)	74.58 ± 10.72	73.45 ± 11.84	0.481	73.43 ± 15.06	71.46 ± 12.74	0.453
BMI (kg/m^2^)	23.94 ± 5.03	23.47 ± 3.77	0.499	28.06 ± 6.93	28.60 ± 6.17	0.648
WBC (10^9^/L)	5.87 ± 1.9	5.81 ± 1.66	0.806	6.85 (5.50,8.00)	6.65 (5.12,7.23)	0.224
Hemoglobin (g/dL)	13.49 ± 2.05	12.98 ± 1.77	0.073	14.03 ± 1.62	13.76 ± 1.60	0.325
PLT (10^9^/L)	198.00 (153.00,243.00)	197.00 (148.50,223.00)	0.849	236.50 (201.25,286.75)	210.00 (152.00,264.75)	0.011
Creatinine (μmol/L)	67.40 (56.50,80.00)	72.60 (60.35,89.95)	0.081	77.79 (63.20,90.17)	89.28 (72.71,112.27)	0.008
eGFR (mL/min/1.73m^2^)	89.96 (76.30,103.88)	84.48 (67.37,96.07)	0.023	85.74 (66.49,108.19)	72.17 (57.17,88.06)	0.004
TC (mmol/L)	4.68 ± 0.91	4.88 ± 0.97	0.114	5.06 ± 0.94	4.75 ± 1.34	0.155
LDL‐C (mmol/L)	2.61 ± 0.75	2.79 ± 0.84	0.101	2.91 (2.35,3.46)	2.44 (1.97,3.48)	0.084
HDL‐C (mmol/L)	1.31 ± 0.3	1.36 ± 0.32	0.238	1.50 ± 0.48	1.39 ± 0.47	0.172
Triglyceride (mmol/L)	1.27 (0.96,1.82)	1.21 (0.94,1.81)	0.775	1.20 (0.91,1.63)	1.21 (0.83,1.76)	0.746
FBG (mmol/L)	5.84 ± 2.03	5.49 ± 1.60	0.202	6.02 ± 1.37	6.04 ± 1.12	0.940
HbA1c (%)	5.97 ± 0.91	6.18 ± 1.15	0.127	5.74 ± 0.79	5.95 ± 0.65	0.114
HGI (%)	−0.17 (−0.40,0.20)	0.13 (−0.27,0.49)	0.002	−0.10 (−0.33,0.16)	0.05 (−0.14,0.23)	0.026
Uric acid (μmol/L)	282.70 (240.00,336.60)	294.90 (237.95,348.80)	0.554	345.00 (279.60,392.60)	345.00 (279.60,400.00)	0.641
Hypertension (%)	54 (31.6)	42 (64.6)	< 0.001	76 (50.7)	34 (77.3)	0.002
Diabetes mellitus (%)	18 (10.5)	11 (17.5)	0.153	23 (15.3)	12 (27.3)	0.184
Hyperlipidemia (%)	35 (21.1)	24 (39.3)	0.005	55 (42.0)	27 (61.4)	0.016
Cancer (%)	4 (2.3)	3 (4.6)	0.632	32 (21.3)	10 (22.7)	0.844
Chronic pulmonary disease (%)	34 (19.7)	19 (28.8)	0.129	26 (17.3)	10 (22.7)	0.418
Arthritis (%)	94 (54.34)	46 (69.7)	0.031	95 (63.3)	34 (77.3)	0.085

Abbreviations: BMI, body mass index; DBP, diastolic blood pressure; eGFR, estimated glomerular filtration rate; FBG, fasting blood glucose; HDL, high‐density lipoprotein cholesterol; HGI, hemoglobin glycation index; LDL‐C, low‐density lipoprotein cholesterol; PLT, platelet; PP, pulse pressure; SBP, systolic blood pressure; TC, total cholesterol; WBC white blood cell.

Baseline characteristics stratified by HGI tertiles were presented in Table 2. In CHARLS, significant differences across tertiles were observed for PP, WBC, PLT, FBG and HbA1c; higher HGI tertiles were also associated with greater prevalences of diabetes and hyperlipidemia (*p* < 0.05). In NHANES, significant differences across tertiles were observed for hemoglobin, PLT, HDL‐C and HbA1c; higher HGI tertiles were associated with higher prevalences of diabetes and hyperlipidemia (*p* < 0.05).

**Table 2:** Baseline demographic and clinical characteristics stratified by HGI tertiles in the CHARLS and NHANES cohort.

	CHARLS				NHANES			
	Tertile 1 (*n* = 88)	Tertile 2 (*n* = 87)	Tertile 3 (*n* = 89)	*p*	Tertile 1 (*n* = 63)	Tertile 2 (*n* = 65)	Tertile 3 (*n* = 66)	*p*
Age (years)	63.84 ± 10.74	63.74 ± 9.67	65.60 ± 10.06	0.394	68.02 ± 13.04	68.50 ± 11.77	68.00 ± 11.00	0.964
Male sex (%)	52 (59.1)	37 (42.5)	34 (38.2)	0.013	27 (42.9)	32 (49.2)	30 (45.5)	0.672
Education (%)				0.657				0.784
Below high school	72 (81.8)	70 (80.5)	70 (78.7)		23 (36.5)	26 (40.0)	23 (34.9)	
High school and above	16 (18.2)	17 (19.5)	19 (21.3)		40 (63.5)	39 (60.0)	43 (65.2)	
Smoke (%)	47 (53.4)	28 (32.2)	39 (43.8)	0.018	32 (50.8)	39 (60.0)	45 (68.2)	0.104
SBP (mmHg)	124.14 ± 19.33	127.09 ± 21.13	128.20 ± 20.85	0.409	132.01 ± 21.75	130.95 ± 22.41	123.20 ± 17.38	0.051
DBP (mmHg)	74.85 ± 11.77	75.49 ± 11.78	74.30 ± 13.70	0.820	69.17 ± 16.36	66.13 ± 17.21	63.48 ± 15.17	0.188
PP (mmHg)	77.27 ± 11.53	71.19 ± 10.73	74.47 ± 9.95	0.001	73.32 ± 16.52	71.76 ± 13.59	74.02 ± 13.53	0.689
BMI (kg/m^2^)	23.38 ± 5.24	24.01 ± 3.40	24.06 ± 5.31	0.587	27.61 ± 6.59	27.30 ± 6.00	29.60 ± 7.44	0.119
WBC (10^9^/L)	5.47 ± 1.44	5.91 ± 1.76	6.19 ± 2.18	0.031	6.15 (5.38,7.43)	6.80 (5.88,8.17)	7.15 (5.43,7.95)	0.169
Hemoglobin (g/dL)	13.55 ± 1.93	13.50 ± 1.92	13.04 ± 2.10	0.167	14.31 ± 1.62	14.26 ± 1.37	13.35 ± 1.68	< 0.001
PLT (10^9^/L)	179.00 (135.00,215.00)	200.00 (166.50,239.00)	208.00 (164.00,266.00)	0.005	233.00 (196.75,286.25)	217.50 (176.00,253.25)	248.50 (207.25,295.00)	0.028
Creatinine (μmol/L)	70.60 (58.35,86.18)	67.80 (58.30,77.80)	67.10 (54.80,79.60)	0.191	79.56 (67.58,91.71)	81.33 (67.58,101.22)	78.68 (61.88,95.69)	0.882
eGFR (mL/min/1.73m^2^)	87.06 (72.98,104.10)	87.28 (73.89,98.34)	88.09 (74.89,105.15)	0.529	85.61 (62.35,111.52)	81.91 (63.48,98.15)	83.90 (67.25,106.21)	0.826
TC (mmol/L)	4.61 ± 0.89	4.72 ± 0.81	4.85 ± 1.05	0.215	5.04 ± 0.92	5.11 ± 1.09	4.84 ± 1.12	0.314
LDL‐C (mmol/L)	2.55 ± 0.77	2.66 ± 0.71	2.75 ± 0.83	0.223	2.87 (2.21,3.28)	2.95 (2.32,3.54)	2.70 (2.04,4.39)	0.523
HDL‐C (mmol/L)	1.31 ± 0.34	1.31 ± 0.28	1.35 ± 0.30	0.574	1.62 ± 0.53	1.51 ± 0.48	1.31 ± 0.36	< 0.001
Triglyceride (mmol/L)	1.31 (0.93,1.95)	1.31 (0.98,1.80)	1.13 (0.94,1.61)	0.495	1.10 (0.78,1.68)	1.20 (0.95,1.61)	1.34 (0.96,1.81)	0.201
FBG (mmol/L)	6.26 ± 2.52	5.34 ± 1.20	5.67 ± 1.77	0.006	6.10 ± 1.20	5.87 ± 1.31	6.11 ± 1.43	0.492
HbA1c (%)	5.54 ± 0.69	5.77 ± 0.40	6.73 ± 1.21	< 0.001	5.31 ± 0.41	5.67 ± 0.46	6.37 ± 0.88	< 0.001
HGI (%)	−0.55 (−0.83, −0.37)	−0.14 (−0.20, −0.04)	0.49 (0.27,0.85)	< 0.001	−0.43 (−0.59, −0.30)	−0.08 (−0.13, 0.02)	0.31 (0.18,0.56)	< 0.001
Uric acid (μmol/L)	300.00 (252.20,345.75)	282.70 (234.90,336.60)	282.70 (234.90,330.50)	0.338	333.10 (261.70,392.60)	359.85 (285.50,422.30)	327.10 (281.08,386.60)	0.342
Hypertension (%)	27 (35.5)	34 (42.0)	35 (44.3)	0.516	35 (55.6)	36 (55.4)	39 (59.1)	0.876
Diabetes mellitus (%)	3 (4.0)	8 (9.9)	18 (23.4)	< 0.001	7 (11.1)	4 (6.2)	24 (36.4)	< 0.001
Hyperlipidemia (%)	10 (13.9)	25 (32.1)	24 (31.2)	0.018	17 (27.0)	29 (44.6)	36 (60.0)	0.005
Cancer (%)	1 (1.3)	1 (1.2)	5 (6.3)	0.202	13 (20.6)	16 (24.6)	13 (19.7)	0.726
Chronic pulmonary disease (%)	15 (19.7)	16 (19.5)	22 (27.2)	0.414	9 (14.3)	11 (16.9)	16 (24.2)	0.309
Arthritis (%)	38 (50.0)	47 (57.3)	55 (67.9)	0.072	40 (63.5)	44 (67.7)	45 (68.2)	0.709
Outcome (%)	16 (18.2)	19 (21.8)	32 (36.0)	0.016	8 (12.7)	19 (29.2)	17 (25.8)	0.063

Abbreviations: BMI, body mass index; DBP, diastolic blood pressure; eGFR, estimated glomerular filtration rate; FBG, fasting blood glucose; HDL, high‐density lipoprotein cholesterol; HGI, hemoglobin glycation index; LDL‐C, low‐density lipoprotein cholesterol; PLT, platelet; PP, pulse pressure; SBP, systolic blood pressure; TC, total cholesterol; WBC white blood cell.

### 3.2 Associations Between HGI and CVD

In the CHARLS cohort, patients in the highest HGI tertile exhibited the highest prevalence of CVD in the unadjusted model (Model 1). This association remained statistically significant after full multivariable adjustment (Model 3: OR: 3.28, 95% CI: 1.33–8.08). When HGI was analyzed as a continuous variable, each 1‐unit increase was also significantly associated with higher odds of prevalent CVD.

In the NHANES cohort, after incorporation of the complex survey design, the tertile‐based associations remained non‐significant across all models. However, when HGI was treated as a continuous variable, a significant positive association with CVD prevalence persisted (Model 1: OR: 1.96, 95% CI: 1.42–2.71), and were further attenuated in the fully‐adjusted model (shown in Table 3). Unweighted analyzes yielded qualitatively similar directional patterns and were presented in Table S1.

**Table 3:** Odds ratios for prevalence of CVD across HGI tertiles in the NHANES (weighted) and CHARLS cohorts.

	Model 1		Model 2		Model 3	
	OR (95%CI)	*p*	OR (95%CI)	*p*	OR (95%CI)	*p*
CHARLS						
HGI (per unit increase)	1.73 (1.20,2.49)	0.003	1.68 (1.15,2.44)	0.007	1.93 (1.12,3.33)	0.018
Tertile 1	Reference		Reference		Reference	
Tertile 2	1.26 (0.60,2.64)	0.546	1.30 (0.61,2.76)	0.501	1.18 (0.46,3.02)	0.726
Tertile 3	2.53 (1.26,5.05)	0.009	2.49 (1.23,5.07)	0.012	3.28 (1.33,8.08)	0.010
NHANES						
HGI (per unit increase)	1.96 (1.42,2.71)	0.024	1.87 (1.80,1.95)	0.003	1.11 (0.19,6.31)	0.595
Tertile 1	Reference		Reference		Reference	
Tertile 2	2.38 (0.45,12.63)	0.154	2.57 (0.46,14.49)	0.143	2.49 (0.24,25.63)	0.235
Tertile 3	2.25 (0.25,19.92)	0.251	2.40 (0.11,52.24)	0.345	1.89 (0.10,62.98)	0.517

*Note:* Model 1: unadjusted; Model 2: adjusted for age, sex, education; Model 3: adjusted for age, sex, education, smoke, hypertension, diabetes, hyperlipidemia and eGFR.

### 3.3 Age‐Stratified Associations

Subgroup analyzes stratified by age (≤ 65 *vs.* > 65 years) were shown in Table 4; corresponding baseline characteristics were summarized in Table S2. In CHARLS, among patients aged ≤ 65 years, the highest HGI tertile was significantly associated with higher CVD prevalence after multivariable adjustment, whereas no statistically significant association was observed among those aged > 65 years. In NHANES, an opposite pattern was observed: among patients aged > 65 years, the second HGI tertile was associated with a higher CVD prevalence, while no significant association was present in the ≤ 65‐year group. Formal interaction tests indicated no significant interaction between HGI and age in either cohort (interaction *p* = 0.316 in CHARLS; interaction *p* = 0.506 in NHANES). Given the modest sample sizes, and wide confidence intervals, these stratified findings should be regarded as exploratory.

**Table 4:** Subgroup analyzes for the relationships between HGI and prevalence of CVD under multivariable logistic analyzes.

	Age ≤ 65 years		Age > 65 years	
	OR (95%CI)	*p*	OR (95%CI)	*p*
CHARLS				
HGI (per unit increase)	3.07 (1.14,8.23)	0.027	1.51 (0.78,2.93)	0.227
Tertile 1	Reference		Reference	
Tertile 2	0.94 (0.24,3.63)	0.924	1.34 (0.31,5.73)	0.691
Tertile 3	4.79 (1.29,17.72)	0.019	2.59 (0.65,10.41)	0.179
NHANES				
HGI (per unit increase)	1.91 (0.43,8.56)	0.400	1.04 (0.43,2.52)	0.939
Tertile 1	Reference		Reference	
Tertile 2	3.41 (0.28,42.06)	0.338	3.35 (1.00,11.20)	0.049
Tertile 3	1.94 (0.20,18.93)	0.569	1.25 (0.33,4.71)	0.738

*Note:* Adjusted for age, sex, education, smoke, hypertension, diabetes, hyperlipidemia and eGFR.

### 3.4 Sensitivity Analyzes

Sensitivity analyzes restricted to participants without diabetes (Table S3) yielded results that were broadly consistent with the primary analyzes yet differed in pattern between the two cohorts. In CHARLS, the highest HGI tertile remained significantly associated with higher CVD prevalence (OR: 2.92, 95% CI: 1.21–7.06). In NHANES, a significant association was observed only for the intermediate tertile (OR: 3.01, 95% CI: 1.01–9.07); the association for the highest tertile was attenuated and non‐significant. When HGI was examined as a continuous variable, the positive association persisted in CHARLS but was attenuated in NHANES. To reduce the risk of overfitting, the primary analyzes were repeated with a more parsimonious set of covariates. In CHARLS, the association between the highest HGI tertile and CVD remained significant (OR: 2.50, 95% CI: 1.11–5.64; *p* = 0.027), as did the continuous HGI association (OR: 1.59, 95% CI: 1.00–2.52; *p* = 0.048). In the survey‐weighted NHANES analyzes, the continuous association remained evident (OR: 1.90, 95% CI: 1.07–3.36; *p* = 0.045), while tertile‐based associations were attenuated, consistent with the fully adjusted primary results. Finally, a sensitivity analysis in CHARLS that employed a more restricted CVD definition (excluding the nonspecific category ‘other heart problems’) showed that the highest HGI tertile was significantly associated with CVD only in the unadjusted model; after multivariable adjustment, the tertile associations were attenuated and non‐significant. In contrast, the continuous HGI association remained positive across all models (Table S4). Collectively, these sensitivity analyzes supported the robustness of the observed associations within the limits imposed by the modest sample size, while underscoring the heterogeneous patterns between the two cohorts.

## 4 Discussion

To our knowledge, this is the first study to investigate the association between HGI and prevalent CVD specifically among middle‐aged and older patients with a history of hip fractures, utilizing data from both NHANES and CHARLS. We demonstrated that higher HGI levels were significantly associated with a greater prevalence of CVD in the CHARLS cohort. In the NHANES cohort, after accounting for the complex survey design, the associations for HGI tertiles were attenuated and no longer statistically significant; however, a significant positive association persisted when HGI was treated as a continuous variable. Sensitivity analyzes restricted to participants without diabetes and those using more parsimonious covariate sets yielded broadly consistent patterns within each cohort, while further underscoring the differences between populations. Collectively, these findings indicated a significant association between elevated HGI and prevalent CVD in the Chinese cohort, whereas the evidence from the U.S. cohort is more limited and primarily driven by the continuous measure.

Hip fracture constitutes a major public‐health challenge in aging societies worldwide [18], and is associated with substantial mortality and socioeconomic burden [19]. Individuals with diabetes have a well‐documented elevation in fracture risk [20], particularly at the hip [21], and CVD frequently coexists with osteoporosis and fracture, contributing to adverse outcomes [22]. Emerging evidence also points to bidirectional interactions between bone‐derived hormones (e.g., osteocalcin and lipocalin‐2) and cardiometabolic pathways [23], reinforcing the clinical relevance of cardiovascular assessment in patients who have sustained a hip fracture.

Increasing attention has focused on the interplay between bone and glucose metabolism [24]. Bone functions as an active endocrine organ that secrets multiple bone‐derived molecules involved in insulin resistance and glucose homeostasis [25, 26], while chronic hyperglycemia and advanced‐glycation end‐product accumulation can impair bone formation, accelerate resorption, and raise fracture susceptibility [27]. Within this context, long‐term glycation burden may be relevant to the bone–glucose–vascular axis in patients with hip fracture.

Although HbA1c remains the standard marker of long‐term glycemic control, its accuracy can be influenced by multiple factors, including erythrocyte lifespan, intracellular glucose transport, enzymatic activity, oxidative stress, genetic background, and race/ethnicity [28]. HGI was developed to quantify inter‐individual variation in hemoglobin glycation beyond mean glucose levels and has been reported to capture glycation heterogeneity more effectively than HbA1c or fasting glucose alone [29]. Prior studies have linked higher HGI to CVD in patients with pre‐diabetes or diabetes [12] and established coronary artery disease [30]. The present results extended these observations to middle‐aged and older adults with hip fracture, but only partially: a robust association was evident in CHARLS, whereas findings in NHANES were weaker.

Several mechanisms may underlie the observed association between elevated HGI and CVD. Higher HGI has been correlated with adverse lipid profiles [31], systemic inflammation [32], arterial stiffness [33], and insulin resistance [13]—factors that may promote endothelial dysfunction and atherosclerosis independent of ambient glucose levels [34]. Advanced‐glycation end products themselves can contribute to vascular injury [35]. These mechanisms remain hypotheses generated by prior literature and are not established by the present cross‐sectional data. More basic research was required to elucidate the specific mechanisms.

Age‐stratified analyzes revealed divergent patterns, which must be interpreted with substantial caution. In CHARLS, the association appeared stronger among participants aged ≤ 65 years, whereas in NHANES a signal was observed mainly among those aged > 65 years. This discrepancy may reflect differences in inter‐ethnic variation in bone mineral density, differing burdens of cardiometabolic comorbidity, and unmeasured genetic, dietary, or environmental influences, or reduced statistical power after sample restriction [36, 37]. Given the modest sample sizes, wide confidence intervals, multiple comparisons, and lack of a consistent exposure–response relationship, any biological explanations for the cohort‐ or age‐related differences remain speculative and should be regarded as exploratory rather than evidence of a reproducible age‐dependent effect.

Interestingly, in the NHANES cohort, the association between HGI and CVD was significant in the unadjusted and minimally adjusted models but were no longer statistically significant after full multivariable adjustment. Several factors may account for the attenuated findings in NHANES after survey weighting. The analytic sample of individuals with hip fracture was modest, and the incorporation of sampling weights, stratification, and clustering further reduce effective sample size and effective number of events while combined with the relatively large number of covariates in the fully adjusted model. Categorization into tertiles discards within‐category information and diminishes power, whereas continuous modeling preserves more information and is generally more sensitive to linear or approximately linear associations. Despite the loss of statistical significance for tertiles, the direction of the point estimates in NHANES remained aligned with those observed in CHARLS.

Notably, the association between HGI and CVD persisted in the non‐diabetic subpopulation, indicating that the observed relationships were not solely driven by diabetes status. Taken together, the findings supported a positive association between higher HGI and prevalent CVD in the CHARLS cohort and a more limited, primarily continuous association in NHANES. Given the cross‐sectional design, modest sample sizes, and heterogeneous results across populations, these observations should be viewed as hypothesis‐generating. Prospective studies with larger event numbers are needed to clarify whether HGI is associated with incident cardiovascular events in middle‐aged and older adults with hip fracture.

Several limitations should be acknowledged. First, both hip fracture and CVD were ascertained by self‐report in CHARLS and NHANES, introducing the possibility of recall bias and misclassification. The CHARLS definition included the nonspecific category “other heart problems,” which may have increased heterogeneity relative to the more specific NHANES definition and restrict direct comparability. Although a sensitivity analysis that restricted the CHARLS outcome supported the continuous association, residual misclassification and the consequent reduction in event numbers limit the precision of categorical estimates. In addition, HGI is a mathematically derived residual that quantifies variation in HbA1c after accounting for concurrent fasting glucose; it is not an independent biological marker. Residual confounding by factors that simultaneously affect both HbA1c and glucose metabolism therefore cannot be excluded, and HGI should be interpreted strictly as a complementary rather than a standalone indicator. Second, the analytic samples were modest in NHANES after incorporation of the survey design, and the effective sample size within age strata and certain sensitivity subsets became too small to yield stable weighted estimates. Consequently, age‐stratified analyzes and most sensitivity analyzes were presented as unweighted exploratory results. These secondary findings should be interpreted with caution and were intended to complement the primary weighted analyzes. Additionally, the linear‐regression equations used to derive predicted HbA1c were fitted within these relatively small samples of participants with hip fracture; while this approach improves population specificity, the stability of the coefficients may be lower than that of equations derived from larger reference populations. The findings should therefore be regarded as exploratory. Third, although a broad range of covariates was adjusted for, several clinically relevant variables—including history of falls and use of bone‐protective medications—were unavailable, leaving the possibility of residual confounding. The modest number of events relative to the number of covariates in the fully adjusted models also raised concern about overfitting; sensitivity analyzes that employed more parsimonious models yielded consistent results and thereby reinforced the primary conclusions. Nevertheless, limited power remains a constraint, especially for the NHANES analyzes and for age‐stratified comparisons. Finally, the cross‐sectional design and reliance on self‐reported outcomes preclude causal inference and any assessment of temporal sequence. Because the study examined prevalent rather than incident CVD, statements concerning prediction, risk stratification for future events, or prevention cannot be supported by the present data. Large‐scale prospective studies are required to determine whether HGI is associated with incident cardiovascular events in this population.

## 5 Conclusion

In middle‐aged and older adults with a history of hip fracture, elevated HGI was independently associated with higher prevalence of CVD in the CHARLS cohort and independent of diabetes status. In the NHANES cohort, the tertile‐based associations were attenuated and non‐significant after survey weighting, although a positive association remained when HGI was modeled continuously. These heterogeneous findings indicated that HGI may serve as a complementary marker of inter‐individual glycation variation in selected populations, but they do not demonstrate a consistent, progressive relationship across cohorts. Given the cross‐sectional design, modest sample sizes, and reliance on self‐reported outcomes, the results should be interpreted as hypothesis‐generating. Prospective studies with larger numbers of incident events are needed to clarify whether HGI is associated with future CVD and to evaluate any potential clinical utility and the underlying biological mechanisms.

## Author Contributions

B.Y ontributed to the study concept and design. X.Y.M. and B.Y contributed to data collection. X.Y.M. contributed to the statistical analysis. X.Y.M. and Bo Yuan contributed to the original draft. All authors approved the final version of the manuscript.

## Funding

The authors have nothing to report.

## Ethics Statement

This study was approved by the ethics committee of Peking University (IRB00001052‐11015) and the NCHS Research Ethics Review Board (ERB). The participants provided written consent. All methods were carried out in accordance with the Declaration of Helsinki.

## Consent to Participate

The authors have nothing to report.

## Consent to Publish

The authors have nothing to report.

## Conflicts of Interest

The authors declare no conflicts of interest.

## Supporting information


Supporting File

## Data Availability

The data that support the findings of this study are available from the corresponding author upon reasonable request.
