# Supplementary material for: Association Between Hemoglobin Glycation Index and Prevalence of Cardiovascular Disease in Middle‐Aged and Older Adults With Hip Fracture: Findings From CHARLS and NHANES
Source: Clin Cardiol. 2026 Oct 7;49(10):e70481. doi: 10.1002/clc.70481 (PMC13644551; doi:10.1002/clc.70481)
Supplement: Supplementary file 1 [file CLC-49-e70481-s001.docx]

**Supplemental Table 1** Odds ratios for prevalence of CVD across HGI tertiles in the NHANES (unweighted).

|  | Model 1 | | Model 2 | | Model 3 | |
| --- | --- | --- | --- | --- | --- | --- |
|  | OR (95%CI) | P | OR (95%CI) | P | OR (95%CI) | P |
| HGI (per unit increase) | 1.62 (0.96,2.72) | 0.069 | 1.61 (0.95,2.72) | 0.077 | 1.30 (0.67,2.51) | 0.435 |
| Tertile 1 | Reference |  | Reference |  | Reference |  |
| Tertile 2 | 2.84 (1.14,7.08) | 0.025 | 2.85 (1.12,7.21) | 0.027 | 2.34 (0.82,6.69) | 0.114 |
| Tertile 3 | 2.39 (0.95,6.01) | 0.065 | 2.47 (0.97,6.32) | 0.059 | 1.41 (0.43,4.63) | 0.570 |

Model 1: unadjusted; Model 2: adjusted for age, sex, education; Model 3: adjusted for age, sex, education, smoke, hypertension, diabetes, hyperlipidemia and eGFR.

Supplemental Table 2 Baseline demographic and clinical characteristics stratified by age in the CHARLS and NHANES cohort.

|  | CHARLS | | | NHANES | | |
| --- | --- | --- | --- | --- | --- | --- |
|  | ≤65 (n=143) | >65 (n=121) | P | ≤65 (n=87) | >65 (n=107) | P |
| Age (years) | 56.65±5.68 | 73.55±5.68 | <0.001 | 56.48±5.83 | 77.67±5.19 | <0.001 |
| Male sex (%) | 69 (48.25) | 54 (44.63) | 0.556 | 40 (45.98) | 49 (45.79) | 0.980 |
| Education (%) |  |  | <0.001 |  |  | 0.315 |
| Below high school | 101 (70.6) | 111 (91.7) |  | 31 (35.63) | 41 (38.32) |  |
| High school and above | 42 (29.4) | 10 (8.3) |  | 56 (64.4) | 66 (61.7) |  |
| Smoke (%) | 62 (43.36) | 52 (42.98) | 0.950 | 56 (64.37) | 60 (56.07) | 0.241 |
| SBP (mmHg) | 122.84±19.04 | 130.91±21.29 | 0.002 | 125.44±17.41 | 132.07±23.50 | 0.035 |
| DBP (mmHg) | 76.94±13.24 | 72.38±10.90 | 0.003 | 71.25±13.94 | 61.57±17.18 | <0.001 |
| PP (mmHg) | 74.14±10.77 | 74.49±11.29 | 0.802 | 72.95±14.83 | 73.01±14.38 | 0.977 |
| BMI (kg/m^2^) | 24.67±5.4 | 22.8±3.56 | 0.002 | 30.01±8.3 | 26.6±4.51 | <0.001 |
| WBC (10^9^/L) | 5.84±1.61 | 5.88±2.08 | 0.851 | 6.80 (5.60,8.00) | 6.60 (5.35,7.80) | 0.367 |
| Hemoglobin (g/dL) | 13.82±1.98 | 12.82±1.88 | <0.001 | 14.38±1.44 | 13.64±1.68 | 0.001 |
| PLT (10^9^/L) | 204.00 (161.50,244.00) | 194.00 (147.00,223.00) | 0.063 | 247.00 (208.00,288.50) | 221.00 (180.00,267.50) | 0.009 |
| Creatinine (μmol/L) | 66.50 (57.00,79.30) | 71.10 (58.20,84.10) | 0.151 | 70.72 (60.55,83.10) | 88.40 (70.72,106.52) | <0.001 |
| eGFR (mL/min/1.73m^2^) | 92.06 (81.20,104.24) | 81.09 (67.80,97.57) | <0.001 | 93.95 (83.31,119.48) | 70.59 (52.33,87.97) | <0.001 |
| TC (mmol/L) | 4.78±0.95 | 4.67±0.91 | 0.330 | 4.98±0.93 | 5.01±1.14 | 0.832 |
| LDL-C (mmol/L) | 2.65±0.78 | 2.66±0.78 | 0.918 | 2.85±0.80 | 2.88±0.93 | 0.857 |
| HDL-C (mmol/L) | 1.33±0.28 | 1.31±0.33 | 0.637 | 1.47±0.49 | 1.48±0.47 | 0.930 |
| Triglyceride (mmol/L) | 1.35 (0.97,2.02) | 1.14 (0.94,1.58) | 0.021 | 1.13 (0.86,1.78) | 1.22 (0.90,1.63) | 0.864 |
| FBG (mmol/L) | 5.62±1.49 | 5.91±2.36 | 0.232 | 6.01±1.47 | 6.04±1.19 | 0.893 |
| HbA1c (%) | 5.90±0.82 | 6.16±1.13 | 0.030 | 5.77±0.76 | 5.81±0.76 | 0.756 |
| HGI (%) | -0.17 (-0.37,0.20) | -0.09 (-0.34,0.33) | 0.116 | -0.09 (-0.30,0.15) | -0.07 (-0.29,0.25) | 0.750 |
| Uric acid (μmol/L) | 282.70 (240.00,339.65) | 288.80 (234.90,336.60) | 0.762 | 345.00 (285.50,392.60) | 333.10 (270.65,407.45) | 0.936 |
| Hypertension (%) | 44 (35.77) | 52 (46.02) | 0.109 | 46 (52.87) | 64 (59.81) | 0.332 |
| Diabetes mellitus (%) | 14 (11.48) | 15 (13.39) | 0.657 | 20 (22.99) | 15 (14.02) | 0.049 |
| Hyperlipidemia (%) | 33 (27.73) | 26 (24.07) | 0.530 | 35 (46.67) | 47 (47.00) | 0.870 |
| Cancer (%) | 4 (3.20) | 3 (2.65) | 0.999 | 11 (12.64) | 31 (28.97) | 0.006 |
| Chronic pulmonary disease (%) | 29 (23.02) | 24 (21.24) | 0.741 | 19 (21.84) | 17 (15.89) | 0.289 |
| Arthritis (%) | 73 (57.94) | 67 (59.29) | 0.832 | 61 (70.11) | 68 (63.55) | 0.335 |

BMI body mass index, SBP systolic blood pressure, DBP diastolic blood pressure, PP pulse pressure, eGFR estimated glomerular filtration rate, WBC white blood cell, PLT platelet, TC total cholesterol, LDL-C low-density lipoprotein cholesterol, HDL high-density lipoprotein cholesterol, FBG fasting blood glucose, HGI hemoglobin glycation index.

Supplemental Table 3 Sensitivity analysis of the association between HGI and prevalence of CVD, excluding patients with diabetes.

|  | CHARLS | | NHANES | |
| --- | --- | --- | --- | --- |
|  | OR (95%CI) | P | OR (95%CI) | P |
| HGI (per unit increase) | 1.98 (1.02,3.85) | 0.045 | 2.62 (0.79,6.64) | 0.115 |
| Tertile 1 | Reference |  | Reference |  |
| Tertile 2 | 1.19 (0.49,2.88) | 0.701 | 3.01 (1.01,9.07) | 0.049 |
| Tertile 3 | 2.92 (1.21,7.06) | 0.017 | 1.30 (0.36,4.69) | 0.690 |

Adjusted for age, sex, education, hypertension and hyperlipidemia.

Supplemental Table 4 Association between HGI and a more restricted definition of CVD in the CHARLS cohort (excluding “other heart problems”)

|  | Model 1 | | Model 2 | | Model 3 | |
| --- | --- | --- | --- | --- | --- | --- |
|  | OR (95%CI) | P | OR (95%CI) | P | OR (95%CI) | P |
| HGI (per unit increase) | 1.69 (1.13,2.53) | 0.011 | 1.59 (1.04,2.42) | 0.032 | 1.99 (1.09,3.65) | 0.026 |
| Tertile 1 | Reference |  | Reference |  | Reference |  |
| Tertile 2 | 0.78 (0.34,1.77) | 0.549 | 0.79 (0.34,1.82) | 0.579 | 0.89 (0.33,2.35) | 0.808 |
| Tertile 3 | 2.15 (1.03,4.49) | 0.042 | 1.98 (0.93,4.23) | 0.077 | 2.46 (0.99,6.11) | 0.052 |

Model 1: unadjusted; Model 2: adjusted for age, sex, education; Model 3: adjusted for age, sex, education, smoke, hypertension, diabetes, hyperlipidemia and eGFR.
